# New technologies to introduce a catalytic function into antibodies: A unique human catalytic antibody light chain showing degradation of β‐amyloid molecule along with the peptidase activity

**DOI:** 10.1096/fba.1025

**Published:** 2019-01-29

**Authors:** Emi Hifumi, Hiroaki Taguchi, Eiichi Toorisaka, Taizo Uda

**Affiliations:** ^1^ Research Promotion Institute, Oita University Oita Japan; ^2^ Faculty of Pharmaceutical Sciences Suzuka University of Medical Science Suzuka Japan; ^3^ Faculty of Engineering, Department of Sustainable Engineering Yamaguchi University Yamaguchi Japan; ^4^ Faculty of Engineering, Department of Applied Chemistry Oita University Oita Japan; ^5^ Nanotechnology Laboratory Institute of Systems, Information Technologies and Nanotechnologies (ISIT) Fukuoka Japan

**Keywords:** Alzheimer, beta‐amyloid, catalytic antibody light chain, clue, zinc ion

## Abstract

Since the discovery of a natural catalytic antibody in 1989, many catalytic antibodies targeting peptides, nucleotides, virus and bacterial proteins, and many molecules have been prepared. Although catalytic antibodies should have features superior to non‐catalytic monoclonal antibodies, the reports on catalytic antibodies are far fewer than those on normal (non‐catalytic) antibodies. Nowadays, we can obtain any monoclonal antibody we want, which is not the case for catalytic antibodies. To overcome this drawback of catalytic antibodies, much basic research must be done. As one way to attain this purpose, we have been making a protein bank of human antibody light chains, in which the light chains were expressed, purified, and stored for use in screening against many kinds of antigen, to then get clues to introducing a catalytic function in normal antibodies. As the number of stored light chains in the above protein bank has reached the hundreds, in this study, we screened them against amyloid‐beta (Aβ), which is well‐known as one of the molecules causing Alzheimer's disease. We found two interesting light chains, #7TR and #7GY. The former could degrade both a fluorescence resonance energy transfer‐Aβ substrate and Aβ1‐40 full peptide. In contrast, #7GY, whose sequence is identical to that of #7TR except for the amino acids at the 29th and 30th positions, did not degrade the FRET‐Aβ substrate at all. By using a synthetic substrate, Arg‐pNA, the difference between the chemical features of the two light chains was investigated in detail. In addition, we found that the presence of Zn(II) ion hugely influenced the catalytic activity of the #7TR light chain but not #7GY. Through these facts and the discussion, we propose one of the clues to how to put a catalytic function in a normal (non‐catalytic) antibody.

**Abbreviations**
aa: amino acid
AMC: 7‐amino‐4‐methylcoumarin
Arg‐pNA (R‐pNA): benzoyl‐(DL or L)‐arginine‐*p*‐nitroanilide monohydrochloride
CR: constant region domain
DNP: 2,4‐dinitrophenyl
FRET: fluorescence resonance energy transfer
PBS: phosphate buffered saline
pNA: *p*‐nitroanilide
SDS‐PAGE: sodium dodecyl sulfate polyacrylamide gel electrophoresis
TGT: glycine/Tris buffer containing 0.025% Tween20

## 1 INTRODUCTION

Since the discovery of a natural catalytic antibody in 1989,^1^ many catalytic antibodies targeting peptides,^2^, ^3^ nucleotides,^4^, ^5^, ^6^ virus and bacterial proteins,^7^, ^8^, ^9^, ^10^, ^11^, ^12^ and molecules such as factor VIII,^13^ CCR5,^14^ and TNFα^15^, ^16^ have been established. The cleaving mechanism of the peptide bond of antigens by catalytic antibodies is considered to be similar to that of serine proteases through studies on site‐directed mutagenesis,^17^, ^18^ X‐ray crystallography,^19^ etc.

Recently, molecule‐targeting drugs (as antibody drugs) have been actively developed throughout the world. Mostly, the targeted molecules concern proteins relating to cancer, Alzheimer's, rheumatism, viruses, etc. Monoclonal antibodies against these molecules^20^, ^21^ have been prepared for practical use, mainly in this decade. Although catalytic antibodies should have features superior to those of non‐catalytic monoclonal antibodies, few studies on them have been reported so far.

We can obtain whatever monoclonal antibody we want if it is a normal protein or peptide. A lot of monoclonal antibodies (non‐catalytic) have been produced since the technology was discovered in 1975.^22^ The number is in thousands. On the other hand, the number of reports on catalytic antibodies is far fewer than that of non‐catalytic monoclonal antibodies. At present, the mainstream technology to producing a catalytic antibody is screening from monoclonal antibodies. It is desirable to design the catalytic antibody for the desired antigen, as we have done for monoclonal antibody production. In order to achieve that purpose, we have to develop a new, advanced or evolutionary technology, or algorithm for the next generation, where catalytic antibodies should be designed on a molecular basis. Therefore, as the first step, the authors herein have been making a protein bank of human antibody light chains for the past ten years. At the present time, the number of expressed and purified light chains including mutants has reached hundreds. By screening the protein bank, we can obtain many kinds of catalytic and non‐catalytic antibodies, which will provide us a lot of information on how to design the catalytic antibody we want.

In addition, one has to be aware of the structural diversity problem of antibodies. We were able to solve the structural diversity issue, and reported how to control for an antibody having a mono‐form structure.^23^, ^24^, ^25^ With this technology, we can make the catalytic antibody structure take a monomolecular form, which is necessary to preparing the same conformational antibody to attain effective production well as reproducible reactivity.

In this study, antibody light chains stored in the protein bank have a structure taking a monomolecular form, and they were screened using a fluorescence resonance energy transfer amyloid‐beta (FRET‐Aβ) peptide including a part of sequence of Aβ1‐40 full peptide, which is well‐known as one of the causes of Alzheimer's disease. We found two interesting light chains, #7TR and #7GY, which possessed identical amino acid (aa) sequences except for the 29th and 30th positions in the sequences consisted of 220 residues. The former had catalytic activity but latter did not. Therefore, we examined their chemical features in detail and found one of the clues on how to introduce a catalytic function in a non‐catalytic antibody light chain.

## 2 MATERIALS AND METHODS

### 2.1 Reagents

Chemical reagents such as Tris, glycine, CuCl_2_·2H_2_O, ZnCl_2_, ZnSO_4_·7H_2_O, KCl, Na_2_HPO_4_·12H_2_O, NaCl, and KH_2_PO_4_ were purchased from Wako Pure Chemical Industries Ltd., Osaka, Japan (Guaranteed Reagent). The synthetic substrate peptidyl‐pNA, benzoyl‐(DL or L)‐arginine‐p‐nitroanilide monohydrochloride (Arg‐pNA), was purchased from Peptides Institute Inc, Osaka, Japan. Tryptone and yeast extract were purchased from Becton‐Dickinson and Company, NJ, USA.

### 2.2 Amplification of DNA fragments encoding light chains from germline genes of subgroup

The human kappa light chain gene was prepared in accordance with the criteria described in a reference.^12^ Briefly stated, peripheral blood lymphocytes were harvested from 100 mL of peripheral blood obtained from healthy volunteers. The lymphocytes were harvested using a Ficoll‐Paque gradient medium (GE Healthcare UK Ltd., Buckinghamshire, England) and stored appropriately. Total RNA was prepared from 3.0 × 10^7^ cells using an RNA isolation kit (Stratagene, La Jolla, CA, USA) for synthesizing cDNA. Oligo (dT) was used for reverse transcription PCR using the total RNA as a template (ThermoScript RT‐PCR System; Invitrogen, Carlsbad, CA, USA). DNA fragments encoding human light chains were amplified from the cDNA by PCR using four primers separately as a forward primer (5′‐cacctagGATATTGTGATGACCCAG‐3′, 5′‐cacctagGATATTGTGATGACTCAG‐3′, 5′‐cacctagGATGTTGTGATGACTCAG‐3′, 5′‐cacctagGAGATTGTGATGACCCAG‐3′), and one reverse primer (5′‐ACACTCTCCCCTGTTGAAGCTCTTTGTG‐3′) including a direct insert to the TOPO site and a start codon. The PCR reaction occurred under the following incubation conditions: 5 minutes at 95°C, and 35 cycles of 15 seconds at 95°C, 50 seconds at 54°C for annealing, and 90 seconds at 72°C for extension.

The amplified DNA fragments were separated by 3% agarose gel electrophoresis. Fragments of the expected size were extracted using the QIAquick Gel Extraction Kit (Qiagen, Valencia, CA, USA). Purified PCR products were directly ligated to pET TOPO vectors (Invitrogen), and the vectors were transformed into TOP10 cells continuously with the Champion pET101 Directional TOPO Expression Kit (Invitrogen). After propagation of the vectors in the TOP10 cells, the inserted pET21b(+) vector was repurified and transformed into BL21 Star (DE3) for expression of light chains belonging to subgroup II.

### 2.3 Sequencing and molecular modeling

The #7GY and #7TR clones were sequenced with the ABI automated DNA sequencer Model 3100 (Applied Biosystems, CA, USA) using the universal primer of a T7 promoter. GENETIX Ver. 8 (GENETIX, Tokyo, Japan) software was used for sequence analysis and deduction of aa sequences.

Computational analysis of the antibody structures was performed using the deduced antibody light chain aa sequences by Discovery Studio (Accelrys Software, San Diego, CA, USA). For the homology modeling, the template structures were made by a BLAST search, following the minimization of the total energy of the molecule by using the CHARMM algorithm. The resulting Protein Data Bank (PDB) data were used for modification of the CDR (complementarity‐determining region) structures defined by the Kabat numbering system.

### 2.4 Expression and purification of #7GY and #7TR light chains

PCR‐Blunt II TOPO vectors containing DNA fragments encoding human light chains were digested with restriction enzymes *Nco*I and *Xho*I (New England Biolabs, Beverly, MA, USA). The resulting DNA fragments were inserted between the same restriction sites of expression vector pET20b (+) (Novagen, Madison, WI, USA). To express light chains, the expression vectors were transformed into BL21 (DE3) pLysS (Novagen). The transformant was grown at 37°C in 1 L of a Luria‐Bertani medium containing 100 µg/mL of ampicillin to 0.6 OD at 600 nm and then incubated with 0.01 mmol/L isopropyl‐β‐D‐thiogalactopyranoside (Wako Pure Chemicals, Osaka, Japan) for 20 hours at 18°C.

Cells were harvested by centrifugation (3500 *g*, 4°C, 10 minutes) and then re‐suspended in a solution (0.25 mol/L NaCl, 25 mmol/L Tris‐HCl, pH 8.0). The cells were lysed by ultrasonication for 2 minutes × 3 in an ice bath, followed by centrifugation (14 000 *g*, 4°C, 20 minutes). The expressed human light chain was recovered as the supernatant. A soluble fraction from *Escherichia coli* was directly applied to Ni‐NTA column chromatography (Takara, Otsu, Japan) equilibrated with 50 mmol/L Na phosphate, pH 7.0, containing 300 mmol/L NaCl. The elution was performed by increasing the concentration of imidazole from 0 to 300 mmol/L. Fractions containing 31‐kDa protein (corresponding to a human light chain) in sodium dodecyl sulfate polyacrylamide gel electrophoresis (SDS‐PAGE) analysis were collected, and the protein concentration was determined by the Bradford and Lowry assay method using a DC protein assay kit (Bio‐Rad). In order to make the light chain structure take a monomolecular form, 1.25 equivalent of CuCl_2_ (50 mmol/L solution) to the light chain was added in the solution and allowed to react for 16 hours under the condition of 4°C. The solution was concentrated to 6.0 mg/mL of the light chain by using the Amicon Ultra 10000 (Merck‐Millipore, Burlington, MA, USA). A 0.5 mol/L EDTA was put into the solution to be 50 mmol/L and allowed to react for 1 hour under the condition of 4°C, followed by dialysis against 2 L of phosphate buffered saline (PBS), twice. In the case of occurrence of aggregation, it was centrifuged at 17,800*g* for 20 minutes under the condition of 4°C. Finally, it was filtered using a 0.2 µm membrane filter (Merck‐Millipore) and stored at 4°C or frozen.

### 2.5 Synthesis of FRET substrate for β‐amyloid

The FRET‐Aβ substrate (MCA‐SNKGAIIGK(DNP)rrr‐NH_2_) was synthesized as follows. The Aβ26‐33 sequence with MCA, K(DNP), and (D‐Arg)_3_ was constructed on a Rink amide resin by the standard 9‐fluorenylmethoxycarbonyl protocol.^26^ The peptide resin was treated with trifluoroacetic acid‐H_2_O‐phenol‐thioanisole‐ethanedithiol (82.5:5:5:5:2.5) at room temperature for 2 hours After removal of the resin by filtration, cold diethyl ether was added to the solution to afford a precipitate, which was collected by centrifugation and washed with diethyl ether. The crude peptide was purified by reversed‐phase HPLC (RP‐HPLC) on a Waters DELTA 600 system, incorporating the 2487 UV/visible detector (Waters, Milford, MA). The HPLC‐purified peptide (retention time: 30.23 minutes; Cosmosil type: 5C18‐AR‐2 [4.6 × 250 mm]; milliQ water in 0.05% TFA: Acetonitrile in 0.05% TFA from 90:10 to 30:70 in 60 minutes [1.0 mL/min]) was characterized by ESI‐MS.

### 2.6 Cleavage assays

To avoid contamination in cleavage assays, the glassware, plasticware, and buffer solutions used in this experiment were sterilized as much as possible by heating (180°C, 2 hours), autoclaving (121°C, 20 minutes), or filtration through a 0.20‐µm sterilized filter. The experiments were mostly performed in a biological safety cabinet to avoid airborne contamination.

#### 2.6.1 Hydrolysis of synthetic peptidyl substrates (peptidase activity)

Cleavage of the amide bond linking *p*‐nitroanilide to the C‐terminal aa in Arg(R)‐pNA substrates (Peptides Institute Inc, Osaka, Japan) was measured at 37°C in a glycine/Tris buffer containing 0.025% Tween20 (TGT buffer; pH 7.7) in 96‐well plates (96‐well plate/353075, Becton‐Dickinson, NJ, USA). The purified light chain (20 µL) was mixed with 180 µl of a synthetic substrate, R‐pNA. The final concentrations of the light chain and the substrate were 10‐15 µmol/L and 200 µmol/L, respectively. Para‐nitroaniline released from the substrate catalyzed by the light chains was detected by the measurement of absorbance at 405 nm, while 620 nm was employed as the reference using a microplate reader (Scanlt 3.1 for Multiskan FC, ThermoFisher Scientific, MA, USA). The peptidase activity of catalytic antibodies was estimated from the concentration of released *p*‐nitroaniline.

#### 2.6.2 Hydrolysis of FRET substrate

The FRET‐Aβ substrate (100 µmol/L) was incubated with the purified L‐chain (5 µmol/L) in 10 mmol/L PBS, pH 7.4, at 37°C. Fluorescence was measured periodically at λ_ex_ = 325 nm and λ_em_ = 394 nm on the SpectraMax M5 Microplate Reader (Molecular Devices, CA, USA) for up to 120 hours All measurements were done in duplicate. Rates of hydrolysis (Fu/h) were determined from plots of fluorescence over time.

#### 2.6.3 Hydrolysis of Aβ1‐40

Synthetic Aβ1‐40 (100 µmol/L) was treated with 1,1,1,3,3,3‐hexafluoro‐2‐propanol (TCI, Japan) to eliminate aggregates^27^ and incubated at 37°C with the purified L‐chain (10 µmol/L) in 10 mmol/L PBS, pH 7.4, for 144 hours The reaction mixtures were fractionated by RP‐HPLC (Cosmosil type: 5C18‐AR‐2 [4.6 × 250 mm]; milliQ water in 0.05% TFA: Acetonitrile in 0.05% TFA from 90:10 to 30:70 in 60 min [1.0 mL/min]). The peaks fractionated were analyzed by ESI‐MS.

### 2.7 Kinetics

The concentration of the #7TR light chain was fixed at 9.2 µmol/L and that of the Arg‐pNA substrate was varied from 12.5 to 400 µmol/L at 37°C in the TGT buffer (pH 7.7). The concentration change of the Arg‐pNA substrate within 0.5‐1 hour after mixing the #7TR light chain and the Arg‐pNA substrate was regarded as the initial rate of the reaction.

### 2.8 Fluorescence spectroscopy

The Gly/Tris solution containing 0.025% Tween20 (TGT buffer) was used as the solution. Fluorescence spectra (Fluorescence spectrum FP8300, JASCO, Japan; λ_ex_, 276.6 nm; λ_em_, 250‐450 nm; Bandwidth, 5 nm; Lamp, Xenon; Sample volume, 300 µL in quartz cell) were taken for each sample, such as #7GY, #7TR, and CR (constant region domain), with or without Zn(II) ions.

### 2.9 UV/VIS spectroscopy

Ultraviolet‐visible spectroscopy (UV/VIS) spectra were taken by an MPS‐2400 spectrophotometer (Shimadzu, Kyoto, Japan) in the range of 900‐220 nm with a bandwidth of 1 nm. An aliquot of CuCl_2_ solution (1 eq; 40 µmol/L) was added in the Ni‐NTA eluent and incubated for 20 hours. After the samples were dialyzed against PBS (500 mL, 1 time), the spectra measurement was performed on them.

## 3 RESULTS

### 3.1 Expression and purification

DNA encoding a human light chain was inserted into expression vector pET20b (+), and the light chain was expressed in *E coli* with a C‐terminal histidine tag. After ultrasonication of the *E coli*, a soluble fraction was recovered and applied to Ni‐ion affinity chromatography, as described in Section 2. After this step, Cu(II) ion was added to make the heterogeneous structures of the light chain (possessing different electrical charges) to the homogeneous (a single charge), which contributes to the efficient production and the high reproducibility (see Reference 23, 24). Then, the treatment of EDTA (for taking out Cu from the system) was carried out to keep the stability of the homogeneous structure for a long‐term period. In this study, 61 kinds of light chains prepared as described above were submitted for the screening assays using the FRET‐Aβ substrate. Two unique antibody light chains, #7GY and #7TR (which are belonging to A3/A19 family of subgroup II of human kappa light chain), were focused on and investigated in detail. The results of SDS‐PAGE analysis for #7GY and #7TR under the reduced and non‐reduced conditions are presented in Figure S1, where 51‐ and 26‐kDa bands corresponding to the dimer and monomer, respectively, were observed. Under the reduced condition, only the 31‐kDa band corresponding to the monomer was detected. In this experiment, we could obtain 21.3 mg and 18.3 mg of purified #7GY and #7TR light chains, respectively, from a one‐liter scale culture.

### 3.2 Screening of human catalytic antibodies using FRET‐Aβ substrate

#### 3.2.1 FRET‐Aβ substrate structure

**Figure 1:**
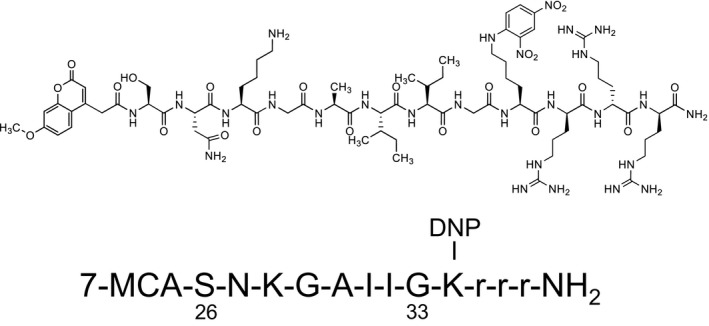
Molecular structure of FRET‐Aβ. The sequence 26‐33 was selected from the Aβ1‐40 peptide. MCA: 4‐methyl‐coumaryl‐7‐amide; DNP: 2,4‐dinitrophenyl; r: Arginine residue that is adducted to elevate the hydrophilic property. FRET‐Aβ substrate has a molecule of MCA at the N‐terminal and a molecule of Lys(DNP) at the C‐terminal of the sequence 26‐33 peptide

We prepared the FRET‐Aβ substrate in accordance with a reference,^28^ which has a part of the sequence from the 26th aa to 33rd aa, SNKGAIIG (8mer), of the amyloid‐β(Aβ)1‐40 full‐size peptide. A molecule of MCA at the N‐terminal and a molecule of Lys(DNP) at the C‐terminal of the SNKGAIIG peptide were introduced to make the FRET‐Aβ substrate. The structure is shown in Figure 1.

#### 3.2.2 Light chains capable of cleaving FRET‐Aβ substrate

**Figure 2:**
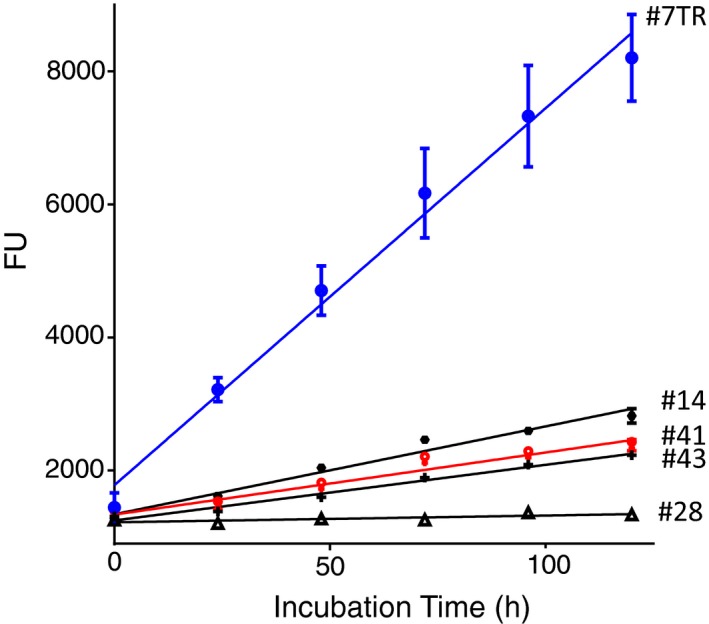
Cleavage for fluorescence resonance energy transfer amyloid‐beta (FRET‐Aβ) substrate. Light chain: 5 µmol/L; FRET‐Aβ: 100 µmol/L; Reaction temperature: 37°C. Sixty‐one purified human light chains were screened using the FRET‐Aβ substrate. #7TR showed the most reactive feature (ΔFu = 57 Fu/hr), while #28 exhibited the lowest activity (ΔFu = 1 Fu/hr)

**Figure 3:**
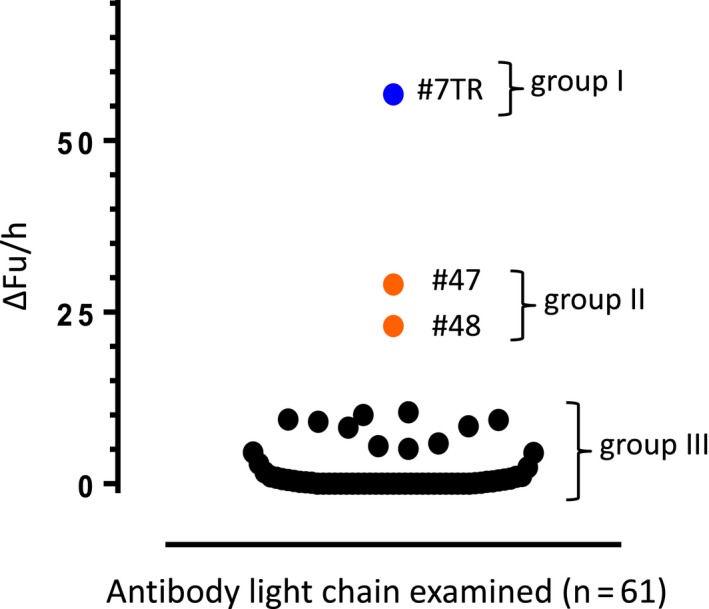
Screening tests for 61 light chains. The #7TR light chain had the highest activity cleaving the fluorescence resonance energy transfer amyloid‐beta substrate, following the #47 and #48 light chains. Other light chains including #7GY did not show catalytic activity

Sixty‐one purified human light chains were screened using the above‐described FRET‐Aβ substrate. The results for several light chains are shown in Figure 2, where 5 µmol/L of a light chain and 100 µmol/L of the FRET‐Aβ substrate in PBS (pH = 7.4) were incubated for 120 hours under the condition of 37°C, which was an experiment done in duplicate. #7TR showed the most reactive feature (ΔFu = 57 Fu/h), while #28 exhibited the lowest activity (ΔFu = 1 Fu/h). The 61 samples screened are summarized in Figure 3, where there are three groups. Group I is the most reactive light chain like #7TR. Group II showed the moderate catalytic activity such as #47 and #48. Third group includes very low or non‐active light chains. #7GY is belonging to group III.

#### 3.2.3 Identification of scissile bond of FRET‐Aβ substrate

**Figure 4:**
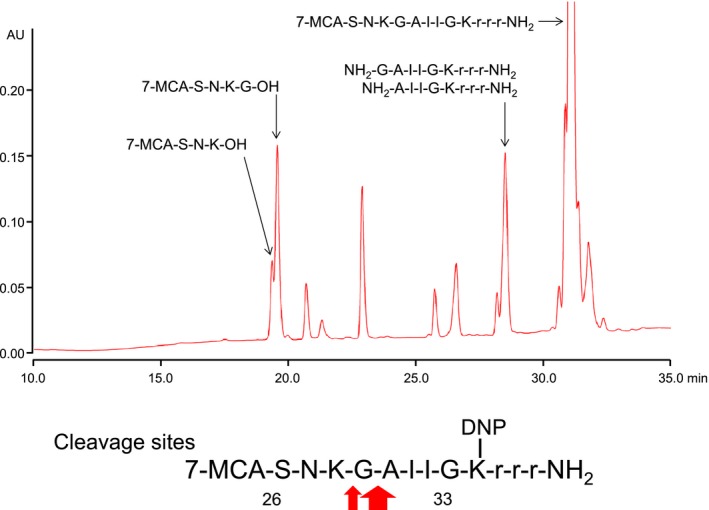
Cleaved peptide bonds of fluorescence resonance energy transfer amyloid‐beta. From both HPLC analysis and mass spectroscopy, several fragments such as MCA‐S‐N‐K‐OH (19.364 minutes), MCA‐S‐N‐K‐G‐OH (19.566 minutes), NH_2_‐A‐I‐I‐G‐K‐r‐r‐r‐NH_2_ (28.185 minutes), and NH_2_‐G‐A‐I‐I‐G‐K‐r‐r‐r‐NH_2_ (28.509 minutes) were detected, indicating that the K28‐G29 and/or G29‐A30 peptide bond was cleaved

As #7TR showed the strong catalytic activity, the reaction products were analyzed by means of HPLC and mass spectroscopy. The results are presented in Figure 4. The fragments such as MCA‐S‐N‐K‐OH (19.364 minutes), MCA‐S‐N‐K‐G‐OH (19.566 minutes), NH_2_‐A‐I‐I‐G‐K‐r‐r‐r‐NH_2_ (28.185 minutes), and NH_2_‐G‐A‐I‐I‐G‐K‐r‐r‐r‐NH_2_ (28.509 minutes) were detected, suggesting that K28‐G29 and/or G29‐A30 were cleaved. As the peak of MCA‐S‐N‐K‐G‐OH is higher than MCA‐S‐N‐K‐OH, #7TR could cleave mainly the G29‐A30 peptide bond.

#### 3.2.4 Difference in features between #7TR and #7GY

#7TR and #7GY have very similar aa sequences. The former has aas of Thr29‐Arg30 at the 29th and 30th positions of the aa sequence. The latter possesses Gly29‐Tyr30 at the same positions. The aa sequences except for these two aas are identical as shown in Figure S2. The #7TR light chain exhibited the high reactivity to cleave the FRET‐Aβ substrate, but #7GY did not at all. These phenomena suggest a crucial factor for an antibody to possess a catalytic function, which will be discussed later.

#### 3.2.5 Cleavage of Aβ1‐40 peptide

**Figure 5:**
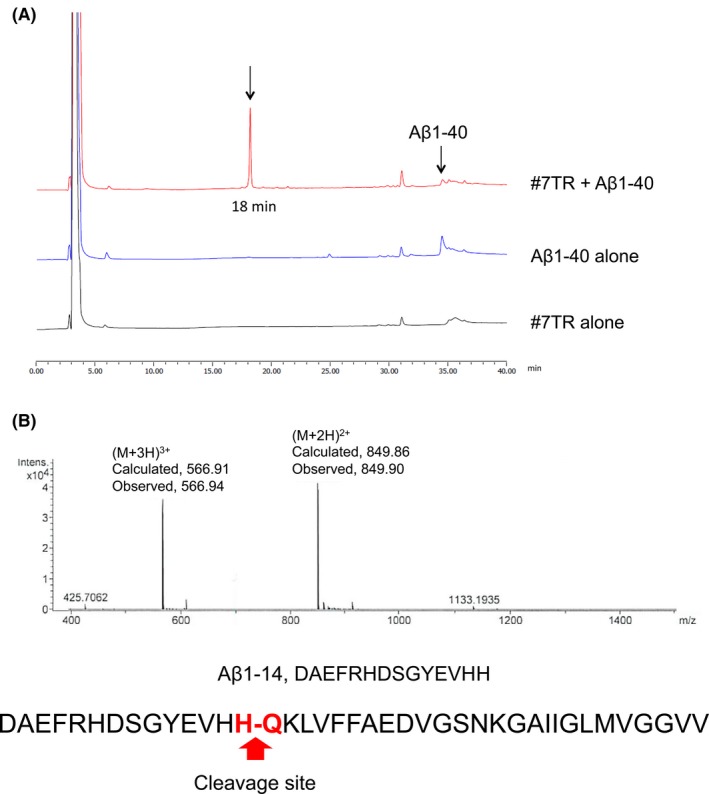
Cleavage of Aβ‐1‐40 peptide. The Aβ1‐40 peptide was incubated with the #7TR catalytic light chain for 144 hours. Light chain: 10 µmol/L; Aβ1‐40 peptide: 100 µmol/L; Reaction temperature: 37°C. A, HPLC analysis. In the case of only the Aβ1‐40 peptide, a peak at the retention time of around 35 minutes was detected. When the Aβ1‐40 peptide was mixed with the #7TR catalytic light chain, a new peak was observed at the retention time of 18 minutes. B, Mass spectroscopic analysis. The peak appearing at the retention time of 18 minutes was analyzed by mass spectroscopy. The divalent mass was detected at 849.9 *m/2z* and the trivalent at 566.94 *m/3z*. This was identified as a fragment, DAEFRHDSGYEVHH, from the Aβ1‐40 peptide, indicating that the H13‐Q14 peptide bond was cleaved

Next, a full Aβ1‐40 peptide was used for a cleavage assay using the #7TR catalytic light chain. In this experiment, 10 µmol/L of the #7TR light chain and 100 µmol/L of the Aβ1‐40 peptide in PBS were incubated for 144 hours under the condition of 37°C. The results are presented in Figure 5A. The peak of the Aβ1‐40 peptide was detected at around 35 minutes. In the reaction products, one clear peak was observed at the retention time of 18 minutes. It was analyzed by mass spectroscopy as shown in Figure 5B. The divalent mass was detected at 849.9 *m/2z* and the trivalent at 566.94 *m/3z*. This was identified as a fragment, DAEFRHDSGYEVHH, from the Aβ1‐40 peptide, indicating that the H13‐Q14 peptide bond was cleaved.

### 3.3 Hydrolysis of synthetic substrate (peptidase activity)

**Figure 6:**
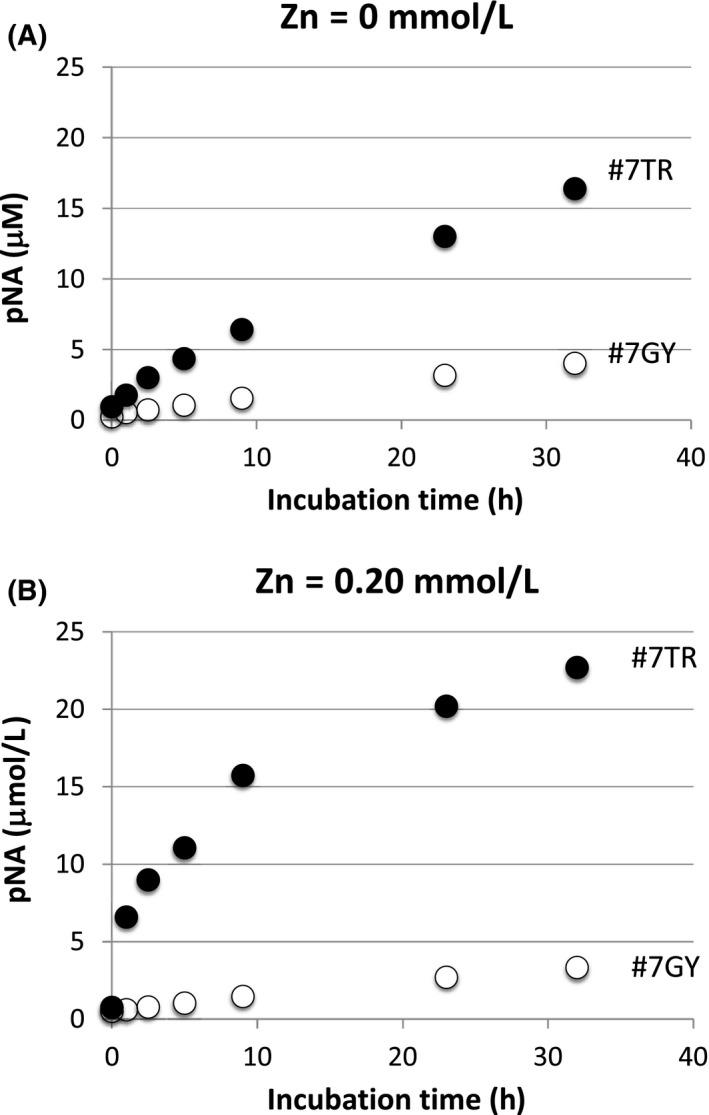
Peptidase activity tests using synthetic substrate (Arg‐pNA). Light chain (#7TR or #7GY): 15 µmol/L; (L)‐Arg‐pNA (R‐pNA) (trypsin‐like substrate): 200 µmol/L; Reaction temperature: 37°C; Reaction volume: 200 µL. In this reaction in the TGT buffer, (L)‐Arg‐pNA was used as the substrate. The reaction was carried out in triplicate. A, Without Zn(II): The #7GY and #7TR light chains cleaved the peptide bond between Arg and pNA in the substrate. The #7TR light chain cleaved the peptide bond with a reaction velocity of 0.63 µmol/L/h at a concentration of 15.0 µmol/L of the light chain. The reaction velocity of #7TR was larger than that of #7GY by a factor of about 3.7‐fold. B, With Zn(II): Concentration of Zn(II) (ZnSO_4_): 0.20 mmol/L. When the Zn(II) ion was added, the cleavage rate was greatly enhanced for #7TR but not for #7GY. The reaction rate of the initial stage became larger than that without Zn(II) by a factor of tenfold. The constant region domain (CR) did not cleave the R‐pNA substrate even under the presence of Zn(II).

Because of the reason stated in Section 3.2.4 *,* we examined the catalytic properties of the two light chains, #7GY and #7TR, using a synthetic Arg (R)‐pNA, which is a trypsin‐like substrate molecule. The results are shown in Figure 6A. Both light chains cleaved the peptide bond between Arg and pNA in the substrate. The #7TR light chain cleaved the peptide bond with a reaction velocity of 0.63 µmol/L/h at a concentration of 15.0 µmol/L of #7TR, while #7GY degraded the R‐pNA with 0.17 µmol/L/h at the same concentration of the light chain. The reaction velocity of #7TR was larger than #7GY by a factor of about 3.7‐fold. The reaction velocity was comparable to those of the 22F6 catalytic light chain (wild type).^12^ On the other hand, the CR domain, which is a common and identical aa sequence in both light chains,^24^ did not cleave the substrate (data not shown).

### 3.4 Effect of Zn(II) ion on hydrolysis of Arg‐pNA substrate

Paul et  al reported the effect of Zn(II) ions on the catalytic activity of their IgV 2E6 catalytic antibody that could cleave an Aβ1‐40/42 peptide.^29^ Therefore, we also investigated the effect of Zn(II) ions in our antibody light chains, #7TR and #7GY. The results are shown in Figure 6B, where the final concentration of ZnSO_4_ was adjusted to 0.20 mmol/L in the reaction solution under the conditions of 200 µmol/L of the R‐pNA substrate and 15 µmol/L of the #7TR or #7GY light chain. When the Zn(II) ion was added, the cleavage rate was greatly enhanced for #7TR but not for #7GY. The cleaved R‐pNA (or released pNA) in 2.5 hours of incubation was 9.0 µmol/L (3.6 µmol/L/h), while it was 0.78 µmol/L (0.31 µmol/L/h) without Zn(II) (Figure 6B). The initial reaction rate in the case with Zn(II) became larger than that without Zn(II) by a factor of tenfold. In the case of the constant domain, no cleavage of R‐pNA was observed regardless of whether the Zn ion was present.

The cleavage reaction of #7TR for R‐pNA under the presence of Zn obeyed the Michaelis‐Menten equation. The values of kcat and Km were 0.022/min and 53.5 × 10^−3^ mol/L, respectively. These values are comparable to those obtained using a human kappa light chain (Bence Jones Protein) taken from a multiple myeloma patient (MOR) as reported by Matsuura.^30^

### 3.5 Concentration of Zn(II) on catalytic reaction

**Figure 7:**
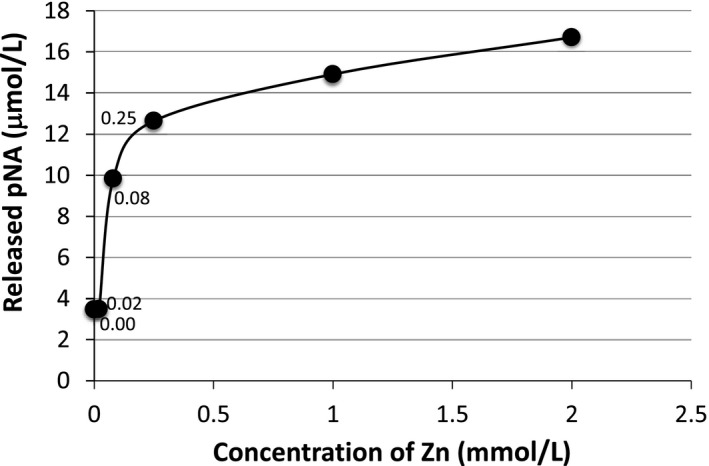
Effect of Zn(II) concentration on catalytic reaction. Light chain (#7TR): 10 µmol/L; Zn(II) ion: 0, 0.02, 0.08, 0.25, 1.0, and 4.0 mmol/L; Reaction time: 6.2 hours. Small content of Zn(II) influenced on the catalytic activity. The cleavage reaction proceeded rapidly from 0.02 mmol/L up to 0.25 mmol/L of Zn ions. Over 0.25 mmol/L, the cleavage reaction reached the maximum. Below 0.02 mmol/L, it was not effective. (The numerical value in the figure represents the Zn(II) concentration)

The Zn(II) ion concentration for the substrate cleavage reaction using the #7TR light chain was examined. The Zn(II) ion concentration was varied from 0 to 2 mmol/L (0, 0.02, 0.08, 0.25, 1.0, and 2.0 mmol/L). The results after an incubation time of 6.2 hours are shown in Figure 7, where 10 µmol/L of #7TR was used. Over 0.02 mmol/L of Zn(II), the cleavage reaction rapidly proceeded up to 0.25 mmol/L of Zn ions. Over 0.25 mmol/L, the reaction seemed to reach the maximum. Below 0.02 mmol/L, it was not effective.

### 3.6 Fluorescent spectra

**Figure 8:**
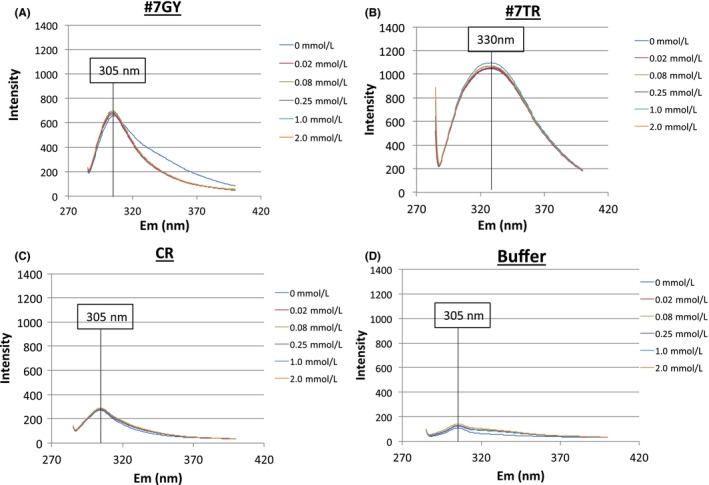
Fluorescent spectra. Concentration of light chains or CR: 4 µmol/L; Zn(II) ion: 0, 0.02, 0.08, 0.25, 1.0, and 2.0 mmol/L; Buffer: TGT (λ_ex_, 276.6 nm; λ_em_, 250‐450 nm) The emission spectra were monitored from 250 to 450 nm. A, #7GY: The emission spectrum was observed at 305 nm. B, #7TR: The emission spectrum was observed at 330 nm, which is quite different from that of #7GY. C, CR (constant region domain): A small emission spectrum was observed at 305 nm, which is the same as observed in the case of #7GY. D, Buffer (TGT): A similar small emission spectrum was observed at 305 nm. For only the #7TR light chain, the maximum emission spectrum was observed at 330 nm and the intensity was strong. For others, the maximum emission spectra appeared at 305 nm. The #7GY and #7TR light chains showed a big difference. This must be due to the different residues, Thr‐Arg and Gly‐Tyr in #7TR and #7GY, respectively. Generally, the spectrum at 330 nm is caused by Try residues, which circumstances were hugely changed by the Thr29 and Arg30 residues

The fluorescence emission spectra for the #7GY, #7TR, and CR domain protein samples were measured with and without the Zn(II) ion. In this experiment, the #7GY, #7TR, and CR concentrations were kept constant at 4 µmol/L. The zinc ion concentration was varied from 0 to 2 mmol/L (0, 0.02, 0.08, 0.25, 1.0, and 2.0 mmol/L). The results are presented in Figure 8A‐D. In these experiments, the emission spectra were monitored from 285 to 400 nm. For #7GY, CR, and TGT buffer (control), the emission maximum at 305 nm was observed. For #7TR, the maximum spectrum appeared at 330 nm and the intensity was strong. The #7GY and #7TR light chains showed a big difference even though their aa sequences were very similar.

In the sequences, there are eight and seven arginine residues in the #7TR and #7GY light chain, respectively. Fifteen and fourteen threonine residues are involved in #7TR and #7GY, respectively. In the CR, there are three arginine and eight threonine residues. Both the #7GY and CR samples did not show any absorbance at 330 nm. Taking these facts and the aa sequences into account, the observed emission maximum of 330 nm for #7TR should be ascribed to the presence of Thr29‐Arg30, which may be interacting each other. Regarding the concentration of Zn(II), neither absorbance at 305 nm nor at 330 nm showed any dependency on the Zn(II) concentration. This suggests that TR does not concern with the binding of Zn.

### 3.7 Molecular modeling

**Figure 9:**
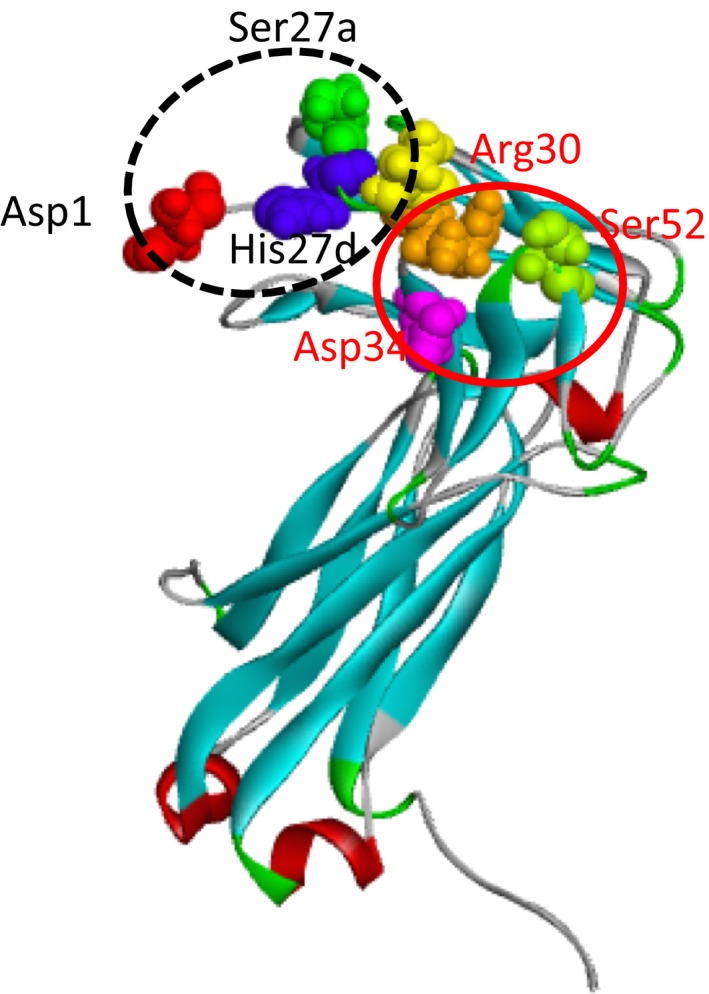
Molecular modeling of light chain structures. By using the deduced amino acid (aa) sequences, the molecular modeling was carried out. Amino acid residues of Asp1, Ser27a, and His27d (or His93) are generally considered to be candidate residues to forming a catalytic triad‐like structure (the black dotted circle indicates the triad). From the model of #7GY and #7TR, 7TR has shorter distances than those of #7GY. (see Table 1). Taking the data on aa sequence, catalytic activity, and spectroscopy into account, a new triad composed of Asp34‐Ser52‐Arg30 may be generated, which is presented as a red circle. As the distance between Thr29‐Arg30 is 3.77 Å, Thr29 may be more preferable than Ser52 in cases

The aa sequences of #7GY and #7TR were deduced from the cDNA sequences. In the results, the two light chains are categorized in A3/A19 of subgroup II. By using the aa sequences, molecular modeling was carried out. Figure 9 shows the structural models of the light chains. Through studies on the catalytic features of antibody light chains so far,^17^, ^18^, ^31^ the aa residues of Asp1, Ser27a, and His27d (or His93) are considered to be candidate residues to forming a catalytic triad‐like structure.

Though the Cα distances are not accurate to estimate the formation of a catalytic triad, it becomes an indicator to consider the possibility. For instance, the residues that the distance is over 15 Å should be excluded from the possibility of the triad. From the model of #7GY and #7TR, the Cα distances among Asp1, Ser27a, and His27d were measured, and they are summarized in Table 1. The distances among Asp1‐Ser27a and His27d‐Asp1 of #7TR are shorter than those of #7GY.

**Table 1:** Distances between Cα of two amino acid residues

	Distance between Cα (Å)		
	Asp1‐Ser27a	Ser27a‐His27d	His27d‐Asp1
#7GY	12.45	8.07	15.89
#7TR	11.26	8.27	14.64
#7TR	Asp34‐Ser52	Ser52‐Arg30	Arg30‐Asp34
	8.35	11.4	11.82

It is well‐known that arginine is a basic aa and can play a role similar to histidine. The #7TR light chain possesses arginine residue at the 30th position in the sequence. The aspartic acid and serine residues closest to Arg30 are Asp34 and Ser52 in the sequence (Figure 9). The Cα distances of Asp34‐Arg30, Arg30‐Ser52, and Ser52‐Asp34 are 11.82, 11.41, and 8.35 Å, respectively, as summarized in Table 1. It should be considered that Thr29 may be involved in formation of the catalytic triad, because the Cα distance between Thr29 and Arg30 is 3.77 Å, which is much shorter than the case of Ser52.

## 4 DISCUSSION

As mentioned in Section 1, we have been preparing a protein bank of human antibody kappa light chains mainly belonging to subgroups I, II, and III. At present, the number of light chains stocked as protein is in hundreds. They are submitted to the assay finding which type of light chain has a catalytic function. In this study, we randomly screened 61 human kappa light chains from the protein bank. In the screening tests using the FRET‐Aβ substrate, three light chains showed catalytic activity to cleave the FRET‐Aβ substrate. Out of them, #7TR exhibited the most reactive feature. In contrast, the #7GY light chain did not show any catalytic activity to degrade the FRET‐Aβ substrate (the data are not shown). Interestingly, the aa sequences of the #7TR and #7GY light chains are very similar except for the residues at the 29th and 30th positions in the sequences. As shown in Figure S2, the aa residues of #7TR are Thr29‐Arg30, and those of #7GY are Gly29‐Tyr30. The aa sequences of the CR are identical. Therefore, we examined the reason why #7TR possesses catalytic features but #7GY does not.

First, we will discuss the phenomena of cleaving the Aβ‐related substrates. In the FRET‐Aβ substrate, we designated the sequence SNKGAIIG from aa26 to aa33 of Aβ as the FRET substrate, because the part of the sequence is important to generating fibrils in the brain. Previously, Taguchi et al found that hydrolysis of Aβ at the Lys28‐Gly29 bond leads to decrease of the formation of Aβ aggregates and protection of neuronal cells from Aβ‐induced neurotoxicity.^32^

In this study, the #7TR light chain cleaved the K28‐G29 and/or G29‐A30 peptide bond of FRET‐Aβ. For the Aβ1‐40 peptide, the light chain preferentially cut the H13‐Q14 peptide bond. The scissile bond was not the same, assuming that the H13‐Q14 peptide bond has a tendency to be easily cleaved, rather than the K28‐G29 and/or G29‐A30 peptide bond in the FRET‐Aβ substrate.

Second, we have to discuss how and why the catalytic function is generated in the case of #7TR, on the basis of molecular level. For this purpose, we employed a synthetic substrate, Arg‐pNA, which is a characteristic substrate for trypsin‐like proteases, as Matsuura et  al^30^ and Ponomarenko et  al^33^ employed the substrate to evaluate catalytic activity. As shown in Figure 6, the #7TR light chain possessed catalytic activity cleaving the substrate, higher than that of #7GY by a factor of 3.7‐fold. CR did not show any catalytic activity at all, indicating that the catalytic site must be present in the variable region. Interestingly, as shown in Figure 7, the Zn(II) ion significantly enhanced the catalytic activity for 7TR, but not for #7GY and CR Considering that #7GY did not cleave the Aβ‐peptide but #7TR did, the residues, Thr29 and Arg30 must be concerned with the recognition of Aβ‐peptide in addition to the catalytic function. To clarify the role of Thr29 and/or Arg30, the mutating experiments replaced by His, Lys, Ser etc are under investigation.

Recently, several groups studying catalytic antibodies have reported the effects of metal ions.^29^, ^34^, ^35^ Especially, Nishiyama et  al found that 225 µmol/L of Zn(II) (or Co(II)) ions exhibited preferable catalytic activity to degrade Aβ1‐40 using their IgV 2E6 catalytic antibody of 20 µg/mL.^29^ From experiments with fluorescence spectroscopy (λ_em_ = 336 nm) and assays of the catalytic activity, they concluded that Zn(II) ions induced the conformational transition and could possess catalytic activity where the preferable catalytic triad consisted of Glu‐Ser‐Arg.

In our experiment, #7TR showed an emission spectrum of 330 nm, but #7GY did not. It is obvious that the emission spectrum observed at 330 nm is ascribed to the presence of Thr29‐Arg30 residues, which might generate a new circumstance around Trp residue (for fluorescence), taking the interaction with Zn ion into account. On the other hand, #7GY and CR showed a small emission spectrum (λ_em_) at 305 nm. Most of the catalytic antibody light chains seem to have a catalytic triad consisting of Asp, Ser, and His. As the chemical features of aa, both histidine and arginine belong to the same category as the basic aa. Namely, arginine residue can replace the function, instead of histidine, and can form a catalytic triad‐like structure. This has been already pointed out by Taguchi et  al.^32^ With respect to the reaction rate reported by Taguchi et  al,^36^ they found 0.052 µM/h/(µM‐Ig) for IEGR‐AMC (IgV_L_‐t′5D3 clone). In our case, #7TR, the rate (without Zn) was 0.06 µM/h/(µM‐L chain) for R‐pNA. These two values of the rate are comparable, suggesting that both catalytic antibodies may have the similar features by using arginine residue instead of histidine residue.^32^ Considering those data and facts, it is preferable that Arg30 (and/or Thr29) should be involved in the formation of the catalytic triad in the #7TR light chain.

**Figure 10:**
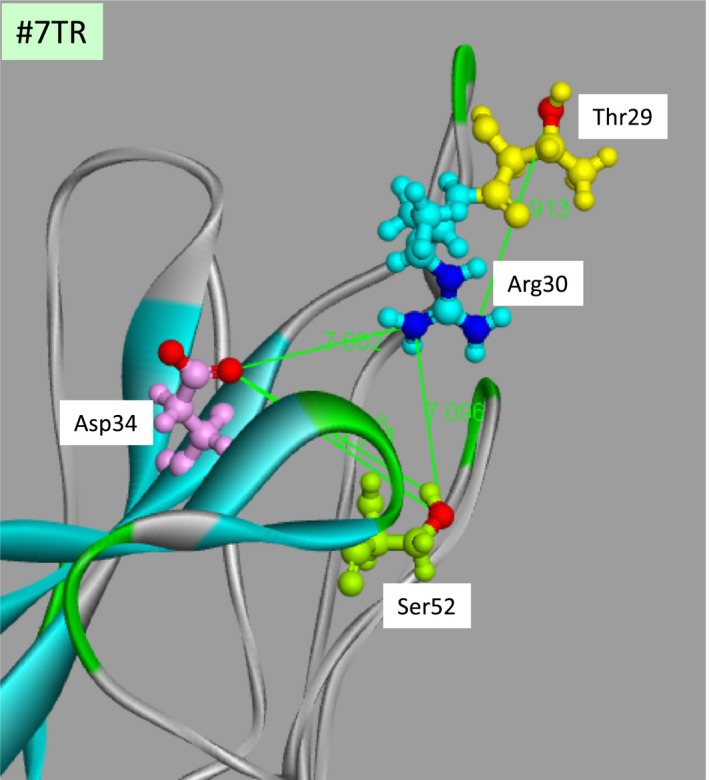
Distances between functional group. The distance of the oxygen (O1) of Asp34 and the nitrogen (N1) of Arg30 is 7.00 Å, and that of the nitrogen (N1) of Arg30 and the oxygen (O) of Ser52 is 7.10 Å. They are longer than those of chymotrypsin (3.00 Å for Asp102(O1)‐His57(N1) and 3.02 Å for His57(N2)‐Ser195(O)) by a factor of about twofold. The Zn(II) ion might bind with the lone pair of nitrogen atoms of Arg, leading to the incident of conformational transition and in turn to a preferable catalytic triad structure. The tremendous enhancement of the cleavage rate of #7TR may derive from this event

The analysis of the molecular modeling indicates the distances between the aa residues relating to catalytic function. An electron relay in the catalytic triad was done with Asp‐Arg‐Ser (or Thr) in #7TR. Furthermore, an oxyanion hole may have been created near the serine residue. Therefore, the distances between Asp‐Arg and Arg‐Ser (or Thr) must be important. The closest aas to Arg30 are Asp34 and Ser52 in all Asp and Ser residues included in the variable region. The distance of the oxygen (O1) of Asp34 and the nitrogen (N1) of Arg30 is 7.00 Å, and that of the nitrogen (N1) of Arg30 and the oxygen (O) of Ser52 is 7.10 Å as shown in Figure 10. They are longer than those of chymotrypsin (3.00 Å for Asp102(O1)‐His57(N1) and 3.02 Å for His57(N2)‐Ser195(O)) by a factor of about twofold. In the presence of Zn(II), the metal ion might bind with the lone pair of nitrogen atoms of Arg (or oxygen atoms of Thr), quickly leading to the incident of conformational transition and in turn to a preferable catalytic triad structure. The huge enhancement of the initial cleavage rate of #7TR is derived from this event.

Through the investigation of chemical and biochemical features of the light chains belonging to A3/A19 family of subgroup II (Kabat's classification), we found “Key aas (Thr29‐Arg30)” which are important to introducing a catalytic function in a non‐catalytic antibody. This first paper concerning to the conversion of a normal antibody to the catalytic antibody by the molecular basis algorithm will accelerate the study on how to easily make a catalytic antibody.

## References

[1] Paul S , Volle DJ , Beach CM , Johnson DR , Powell MJ , Massey RJ . Catalytic hydrolysis of vasoactive intestinal peptide by human autoantibody. Science. 1989;244:1158‐1162. 2727702 10.1126/science.2727702

[2] Mei S , Mody B , Eklund SH , Paul S . Vasoactive intestinal peptide hydrolysis by antibody light chains (communication). J Biol Chem. 1991;266:15571‐15574. 1908455

[3] Hifumi E , Kondo H , Mitsuda Y , Uda T . Catalytic features of monoclonal antibody i41SL1‐2 subunits. Biotechnol Bioeng. 2003;84(7):485‐493. 14574707 10.1002/bit.10806

[4] Shuster AM , Gololobov GV , Kvashuk OA , Bogomolova AE , Smirnov IV , Gabibov AG . DNA hydrolyzing autoantibodies. Science. 1992;256:665‐667. 1585181 10.1126/science.1585181

[5] Parkhomenko TA , Buneva VN , Tyshkevich OB , Generalov II , Doronin BM , Nevinsky GA . DNA‐hydrolyzing activity of IgG antibodies from the sera of patients with tick‐borne encephalitis. Biochimie. 2010;92:545‐554. 20138955 10.1016/j.biochi.2010.01.022

[6] Krasnorutskii MA , Buneva VN , Nevinsky GA . DNase, RNase, and phosphatase activities of antibodies formed upon immunization by DNA, DNase I, and DNase II. Biochemistry. 2011;76:1065‐1072. 22082277 10.1134/S0006297911090124

[7] Hifumi E , Mitsuda Y , Ohara K , Uda T . Targeted destruction of the HIV‐1 coat protein gp41 by a catalytic antibody light chain. J Immunol Methods. 2002;269:283‐298. 12379368 10.1016/s0022-1759(02)00242-9

[8] Paul S , Karle S , Planque S et al. Naturally occurring proteolytic antibodies: selective immunoglobulin M‐catalyzed hydrolysis of HIV gp120. J Biol Chem. 2004;279:39611‐39619. 15269209 10.1074/jbc.M406719200

[9] Hifumi E , Morihara F , Hatiuchi K , Okuda T , Nishizono A , Uda T . Catalytic features and eradication ability of antibody light‐chain UA15‐L against *Helicobacter pylori* . J Biol Chem. 2008;283:899‐907. 17991752 10.1074/jbc.M705674200

[10] Hifumi E , Honjo E , Fujimoto N , Arakawa M , Nishizono A , Uda T . Highly efficient method of preparing human catalytic antibody light chains and their biological characteristics. FASEB J. 2012;26:1607‐1615. 22205784 10.1096/fj.11-195339

[11] Hifumi E , Takao S , Fujimoto N , Uda T . Catalytic and biochemical features of a monoclonal antibody heavy chain, JN1‐2, raised against a synthetic peptide with hemagglutinin molecule of influenza virus. J Am Chem Soc. 2011;133(38):15015‐15024. 21861493 10.1021/ja203922r

[12] Hifumi E , Fujimoto N , Arakawa M et al. Biochemical features of a catalytic antibody light chain, 22F6, prepared from human lymphocytes. J Biol Chem. 2013;288:19558‐19568. 23677996 10.1074/jbc.M113.454579 PMC3707657

[13] Lacroix‐Desmazes S , Sooryanarayana MA , Bonnemain C et al. Catalytic activity of antibodies against factor VIII in patients with hemophilia A. Nature Med. 1999;5:1044‐1047. 10470082 10.1038/12483

[14] Mitsuda Y , Hifumi E , Tsuruhata K , Fujinami H , Yamamoto N , Uda T . Catalytic antibody light chain capable of cleaving a chemokine receptor CCR‐5 peptide with a high reaction rate constant. Biotechnol Bioeng. 2004;86:217‐225. 15052642 10.1002/bit.20031

[15] Hifumi E , Higashi K , Uda T . Catalytic digestion of human tumor necrosis factor‐α by antibody heavy chain. FEBS J. 2010;277:3823‐3832. 20718866 10.1111/j.1742-4658.2010.07785.x

[16] Sharma V , Heriot W , Trisler K , Smider V . A human germ line antibody light chain with hydrolytic properties associated its multimerization status. J Biol Chem. 2009;284:33079‐33087. 19801545 10.1074/jbc.M109.036087 PMC2785149

[17] Gololobov G , Sun M , Paul S . Innate antibody catalysis. Mol. Immunol. 1999;36:1215‐1222. 10684961 10.1016/s0161-5890(99)00141-8

[18] Okochi N , Kato‐Murai M , Kadonosono T , Ueda M . Design of a serine protease‐like catalytic triad on an antibody light chain displayed on the yeast cell surface. Appl Microbiol Biotech. 2007;77:597‐603. 10.1007/s00253-007-1197-0 17899065

[19] Ramsland RA , Terzyan SS , Cloud G et al. Crystal structure of a glycosylated Fab from an IgM cryoglobulin with properties of a natural proteolytic antibody. Biochem J. 2006;396:473‐481. 10.1042/BJ20051739 PMC1462693 16422668

[20] Scott A‐M , Wolchok J‐D , Old J‐J . Antibody therapy of cancer. Nat Rev Cancer. 2012;12:278‐287. 22437872 10.1038/nrc3236

[21] Kotsovilis S , Andreakos E . Therapeutic human monoclonal antibodies in inflammatory diseases. Methods Mol Biol. 2014;1060:37‐59. 24037835 10.1007/978-1-62703-586-6_3

[22] Köhler G , Milstein C . Continuous cultures of fused cells secreting antibody of predefined specificity. Nature. 1975;256:495‐497. 1172191 10.1038/256495a0

[23] Hifumi E , Matsumoto S , Nakashima H et al. A novel method of preparing the mono‐form structure of catalytic antibody light chain. FASEB J. 2016;30:895‐908. 26527062 10.1096/fj.15-276394

[24] Hifumi E , Taguchi H , Kato R , Uda T . Role of the constant region domain in the structural diversity of human antibody light chain. FASEB J. 2017;31:1668‐1677. 28096233 10.1096/fj.201600819R

[25] Hifumi E , Taguchi H , Kato R , Arakawa M , Katayama Y , Uda T . Structural diversity problems and the solving method for antibody light chains In: Boldicke T Antibody Engineering, chapter 10. London, UK: InTech publishing; 2018: 231‐257. ISBN 978‐953‐51‐5294‐1.

[26] Wellings DA , Atherton E . Standard Fmoc protocols. Methods Enzymol. 1997;289:44‐67. 9353717 10.1016/s0076-6879(97)89043-x

[27] Dahlgren KN , Manelli AM , Stine WB Jr , Baker LK , Krafft GA , LaDu MJ . J Biol Chem. 2002;277:32046‐32053. 12058030 10.1074/jbc.M201750200

[28] Baronas‐Lowell D , Lauer‐Fields JL , Borgia JA et al. J Biol Chem. 2004;279(42):43503‐43513. 15292257 10.1074/jbc.M405979200

[29] Nishiyama Y , Taguchi H , Hara M , Planque SA , Mitsuda Y , Paul S . Metal‐dependent amyloid β‐degrading catalytic antibody construct. J Biotech. 2014;180:17‐22. 10.1016/j.jbiotec.2014.03.026 PMC4512298 24698848

[30] Matsuura K , Yamamoto K , Shinohara H . Amidase activity of human Bence Jones proteins. Biochem Biophys Res Commun. 1994;204:57‐62. 7945392 10.1006/bbrc.1994.2425

[31] Uda T , Hifumi E . Super catalytic antibody and antigenase. J Biosci Bioeng. 2004;97:143‐152. 16233607 10.1016/S1389-1723(04)70183-8

[32] Taguchi H , Planque S , Nishiyama Y et al. Autoantibody‐catalyzed hydrolysis of amyloid β peptide. J Biol Chem. 2008;283:4714‐4722. 18086674 10.1074/jbc.M707983200

[33] Ponomarenko NA , Vorobiev II , Alexandrova ES et al. Induction of a protein‐targeted catalytic response in autoimmune prone mice: antibody‐mediated cleavage of HIV‐1 glycoprotein GP120. Biochemistry. 2006;45:324‐330. 16388609 10.1021/bi050675k

[34] Odintsova ES , Zaksas NP , Buneva VN , Nevinsky GA . Metal dependent hydrolysis of β‐casein by sIgA antibodies from human milk. J Mol Recognit. 2010;24:45‐59. 10.1002/jmr.1022 20140974

[35] Bezuglova AM , Konenkova LP , Doronin BM , Buneva VN , Nevinsky GA . Affinity and catalytic heterogeneity and metal‐dependence of polyclonal myelin basic protein‐hydrolyzing IgGs from sera of patients with systemic lupus erythematosus. J Mol Recognit. 2011;24:960‐974. 22038803 10.1002/jmr.1143

[36] Taguchi H , Planque S , Sapparapu G et al. Exceptional amyloid β peptide hydrolyzing activity of nonphysiological immunoglobulin variable domain scaffolds. J Biol Chem. 2008;283:36724‐36733. 18974093 10.1074/jbc.M806766200 PMC2606003

