# Supplementary material for: New technologies to introduce a catalytic function into antibodies: A unique human catalytic antibody light chain showing degradation of β‐amyloid molecule along with the peptidase activity
Source: FASEB Bioadv. 2019 Jan 29;1(2):93–104. doi: 10.1096/fba.1025 (PMC6996398; doi:10.1096/fba.1025)
Supplement: Supplementary file 1 [file FBA2-1-93-s001.pdf]

Supplement materials

Fig S1:The results of SDS-PAGE analysis for #7GY and #7TR

Fig S2: Amino acid sequences of #7TR and #7GY light chains.

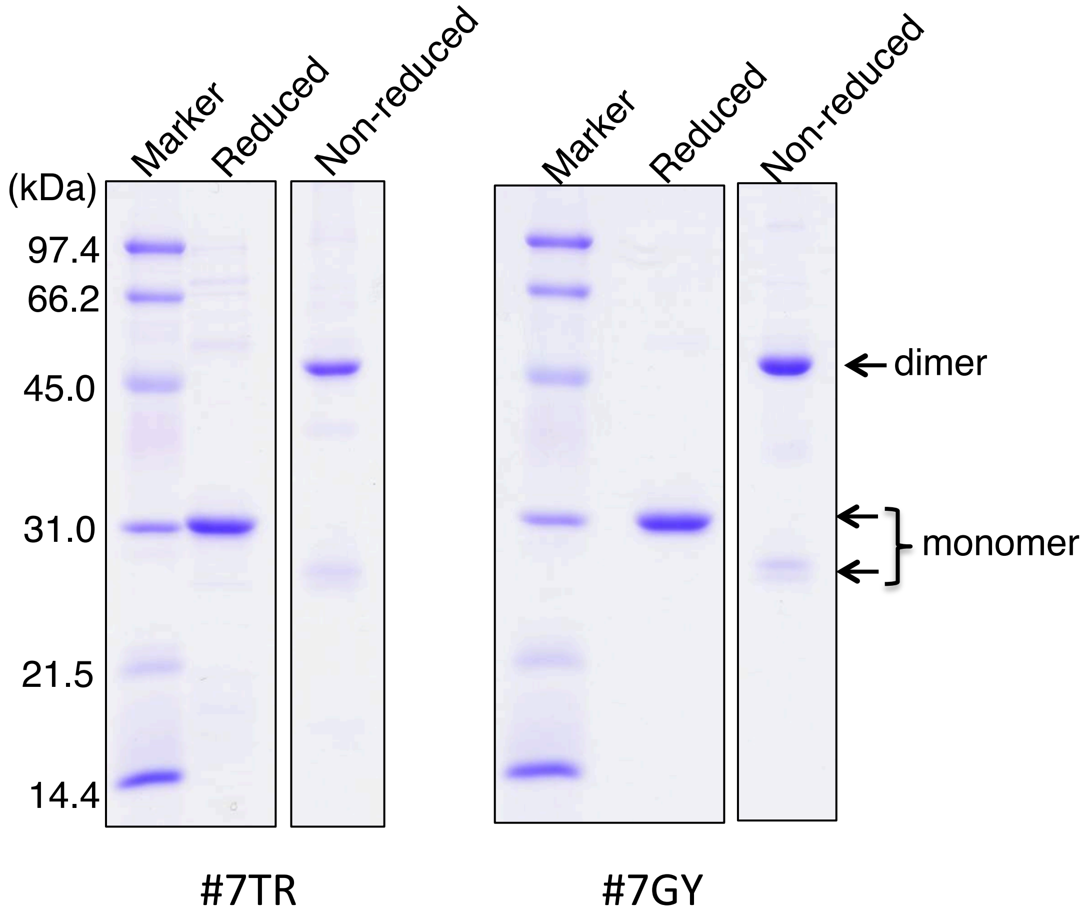
Marker
Reduced
Non-reduced
Marker
Reduced
Non-reduced
(kDa)
97.4
66.2
45.0
31.0
21.5
14.4
dimer
monomer
#7TR
#7GY

Fig. S1

```
#7TR   MDVVMTQSPLSLPVTPGEPASISCRSSQSLLHSNTRNYLDWYLQKPGQSPQLLIYLGSNRASGVPDRFSGSGSG
#7GY   MDVVMTQSPLSLPVTPGEPASISCRSSQSLLHSNGYNYLDWYLQKPGQSPQLLIYLGSNRASGVPDRFSGSGSG
CR
```

Numbering above the first block: 1, 20, 27abcde, 29 30, 40, 60. Labels: CDR-1 (RSSQSLLHSNTRNYLD / RSSQSLLHSNGYNYLD), CDR-2 (LGSNRAS)

Variable region ↓ Constant region

```
#7TR   TDFTLKISRVEAEDVGVYYCMQALQTPRTFGQGTKVEIKRTVAAPSVFIFPPSDEQLKSGTASVVCLLNNFYP
#7GY   TDFTLKISRVEAEDVGVYYCMQALQTPRTFGQGTKVEIKRTVAAPSVFIFPPSDEQLKSGTASVVCLLNNFYP
CR                                            MARTVAAPSVFIFPPSDEQLKSGTASVVCLLNNFYP
```

Numbering above the second block: 80, 100, 120, 140. Label: CDR-3 (MQALQTPR)

```
#7TR   REAKVQWKVDNALQSGNSQESVTEQDSKDSTYSLSSTLTLSKADYEKHKLYACEVTHQGLSSPVTKSFNRGECLEHHHHHH
#7GY   REAKVQWKVDNALQSGNSQESVTEQDSKDSTYSLSSTLTLSKADYEKHKLYACEVTHQGLSSPVTKSFNRGECLEHHHHHH
CR     REAKVQWKVDNALQSGNSQESVTEQDSKDSTYSLSSTLTLSKADYEKHKVYACEVTHQGLSSPVTKSFNRGECLEHHHHHH
```

Numbering above the third block: 160, 180, 200. Label: His-tag (HHHHHH)

Fig. S2
